# Neckline-free open-source cap design and improved motion resistance for perioperative fNIRS procedures

**DOI:** 10.1364/BOE.605391

**Published:** 2026-09-10

**Authors:** Seth Bolton Crawford, Shimra Jaime Fine, Kyla Inez Covato, Caroline Joyce Caveness, Audrey K. Bowden

**Affiliations:** 1 Vanderbilt Biophotonics Center, Vanderbilt University, Nashville, TN 37232, USA; 2 Department of Biomedical Engineering, Vanderbilt University, Nashville, TN 37232, USA; 3 Fine Soft Goods Consulting, Nashville, TN 37076, USA; 4 Department of Mechanical Engineering, Vanderbilt University, Nashville, TN 37232, USA; 5 Department of Electrical Engineering, Vanderbilt University, Nashville, TN 37232, USA

## Abstract

Functional near-infrared spectroscopy (fNIRS) caps are limited by motion artifacts and unstable optode–scalp coupling, which degrade signal quality during extended and naturalistic monitoring sessions. We present a fabric-based, chinstrap-free headcap designed to improve mechanical stability and optical signal fidelity through enhanced coupling and reduced relative motion. The open-source cap incorporates a two-layer design with slip-resistant materials and a circumferential tightening mechanism. Performance was evaluated over a two-hour protocol in a mock operating room environment, with motion quantified via image-based tracking, signal quality assessed using signal-to-noise ratio (SNR), scalp-coupling index (SCI), and breath-holding responses, and user comfort evaluated with a human factors questionnaire. Results show reduced displacement and robust hemodynamic signals, supporting improved reliability of fNIRS measurements in perioperative conditions.

## 1. Introduction

Continuous-wave functional near-infrared spectroscopy (cw-fNIRS) is a non-invasive neuromonitoring technique that measures changes in the concentration of oxygenated and deoxygenated hemoglobin in the outermost layers of the brain cortex [1,2]. A strong advantage of cw-fNIRS compared to other fNIRS formats (e.g., time domain and frequency domain) is that it is relatively inexpensive, portable, and, therefore, can be used to study brain function in natural environments (e.g., healthcare settings, homes, schools, offices) [3,4].

Hospital settings represent one natural environment where fNIRS could be particularly useful [4,5]. Deploying cw-fNIRS across the perioperative window and specifically within the operating room (OR) is of growing interest, as the physiological and neural data it provides could assist in contextualizing adverse postoperative outcomes from numerous surgeries (e.g., cardiac, vascular, head and neck, neurosurgery) [6–8]. Despite this interest, cw-fNIRS adoption in the OR remains limited, as existing systems are primarily designed for well-controlled lab environments and, therefore, suffer from poor signal quality and mechanical instability when implemented in healthcare settings and ORs [9].

For use cases in the perioperative window, minimal physical shifting of the system is necessary to avoid motion artifacts in the data and obtain reliable brain measurements [1–3,10]. While patient motion is primarily an issue before and after surgery, motion artifacts can still manifest in the OR when patients are under anesthesia. For example, during cardiac surgeries, the degree of reclination of the surgical table is frequently adjusted to accommodate surgical access and optimize patient positioning [11,12]. These table movements translate to patient head displacement and can result in corresponding shifts of the fNIRS system.

Additionally, achieving high-quality signal with cw-fNIRS has numerous requirements such as stable system and electronics designs [1,2], proper sensor (both light emitting and detecting components) placement and organization [13,14], sufficient contact between the sensors and the scalp [15], and appropriate data processing techniques [12]. One of the most useful tools for both minimizing motion artifacts and maximizing signal quality is the enclosure used to hold the system in place during data collection. For brain measurements, this enclosure often takes the form of a cap.

Most fNIRS caps are fabric-based, single-layered designs [16,17] with a chinstrap for size adjustment tailored to the wearer and circular openings arranged in agreement with the international 10-10 or 10-20 imaging standards [14] and sized to be compatible with the accompanying fNIRS electronics. However, the soft caps required to achieve this layout and provide comfortable, conformal imaging tends to shift on the heads of wearers during routine movements in the perioperative window [17,18]. These shifts can lead to sensor displacement from targeted brain regions or detachment of the system from the scalp.

In addition to patient movement, the duration of monitoring poses a further challenge to perioperative signal quality. Monitoring before, during, and after surgery often requires multiple hours of data collection, which is substantially longer than sessions typically conducted in controlled lab environments. A review of caps performed by Kassab et al. demonstrated that prolonged monitoring sessions with these caps are susceptible to motion-related artifacts, signal attenuation and reduced patient tolerance [17].

Some research groups have attempted to address problems with cap motion by developing custom cap designs for proper optode registration and specialized data collection. McCann, von Lühmann, and Anaya developed alternative cap designs that can be 3D-printed with elastic resin [13,19,20], and work by Kassab et al. details the development of a custom neoprene cap that was worn by imaging subjects for several hours without reports of user discomfort [21]. However, many of these designs either require MRI or facial scans of the wearer for proper fit or rely on a chinstrap for stabilization, which can limit applicability in healthcare settings. For example, facial scanning technologies may be too costly, bulky, or time-consuming to be deployed within the perioperative window; additionally, for certain surgeries in the OR, such as cardiac surgeries where surgeons require ready access to the neckline, chinstrap-based designs cannot be deployed.

Kassab et al. and Kiguchi et al. suggested that motion resistance could be improved via vacuum and suction-based designs using more elastic materials with a strapless form factor [21,22]. Although not specifically designed for fNIRS, Wellborn et al. developed a motion-resistant suction-based cap for head and neck surgeries [23] using granular jamming, a technique in which granular material is placed within the cap and the air between the granules is removed leaving a tight and conformal fit. However, these designs have yet to be adopted for fNIRS and would likely introduce bulky, wired vacuum systems that could limit portability and complicate data collection in the OR and broader healthcare settings.

With a view towards enabling fNIRS during surgical contexts, we designed a neckline-accessible cap system with improved motion resistance that can be integrated with commercial fNIRS systems. The cap comprises a two-layer design to improve overall motion resistance. The inner first layer is made of a combination of neoprene fabric (3 mm in thickness) and Dycem, which is a medical-grade, latex-free, slip-resistant material. The second layer is the outer layer, made of conformal monofilament fabric (1-mm thick) and functions to secure fNIRS system components, including the optodes and system controller, while preventing wires from protruding and potentially destabilizing the cap. In lieu of a chinstrap, we developed a novel tightening mechanism sandwiched between the layers that spans horizontally across the circumference of the head, enabling a conformal and comfortable fit while maintaining access to the neckline.

We evaluated the cap through motion testing during a data collection session conducted in a mock operating room environment at the Vanderbilt Institute of Surgery and Engineering (VISE). In addition to assessing motion, we evaluated signal quality through hemodynamic data collection and evaluated user comfort through a human factors questionnaire.

To our knowledge, our cap design is the first fNIRS cap to incorporate Dycem, a slip-resistant material that has been used in other medical applications [24], to improve resistance to motion and, thus, motion artifacts. Furthermore, our novel tightening mechanism removes the need for a chinstrap, improving comfort and enabling use in surgical contexts where access to the neckline is required.

While other open-source, commercial system-compatible fNIRS caps exist based on 3D printing, our design is the first fabric-based alternative, which would allow superior scalability (one fabric-based cap can be size adjusted to fit multiple head sizes and shapes, whereas 3D-printed caps are specialized to the anatomy of one person) and improved user comfort (soft contact versus contact with solid, rigid material). Although the cap was developed for use in operating rooms, the achieved results for signal quality, motion resistance, and user comfort also support its applicability for cw-fNIRS data collection in other general settings.

## 2. Methods

### 2.1. System design

Our cap, detailed in Fig. 1, consists of two layers with a novel tightening mechanism in between. The inner layer is primarily made of neoprene fabric, with a two-inch strip of Dycem sewn around the bottom boundary. Neoprene has been used previously in fNIRS cap designs and is particularly useful because of its comfortable and conformal properties. We added Dycem, a slip-resistant, latex-free material, to improve the grip at the cap-scalp interface, so that the cap may maintain a stable, conformal fit and resist displacement during prolonged or dynamic use. Dycem is frequently used in healthcare settings as anti-slipping floor mats in care rooms, wheelchair seat padding to maintain posture, and for wrapping various household objects for arthritic hands. Dycem has also been utilized recently in the design of wearable systems; for example, the material was incorporated into a low-profile ankle exoskeleton to maintain the position of the exoskeleton on the participant’s foot [24]. To our knowledge, our work is the first of its kind to incorporate Dycem into cap systems and for deployment alongside fNIRS.

**Fig. 1.**
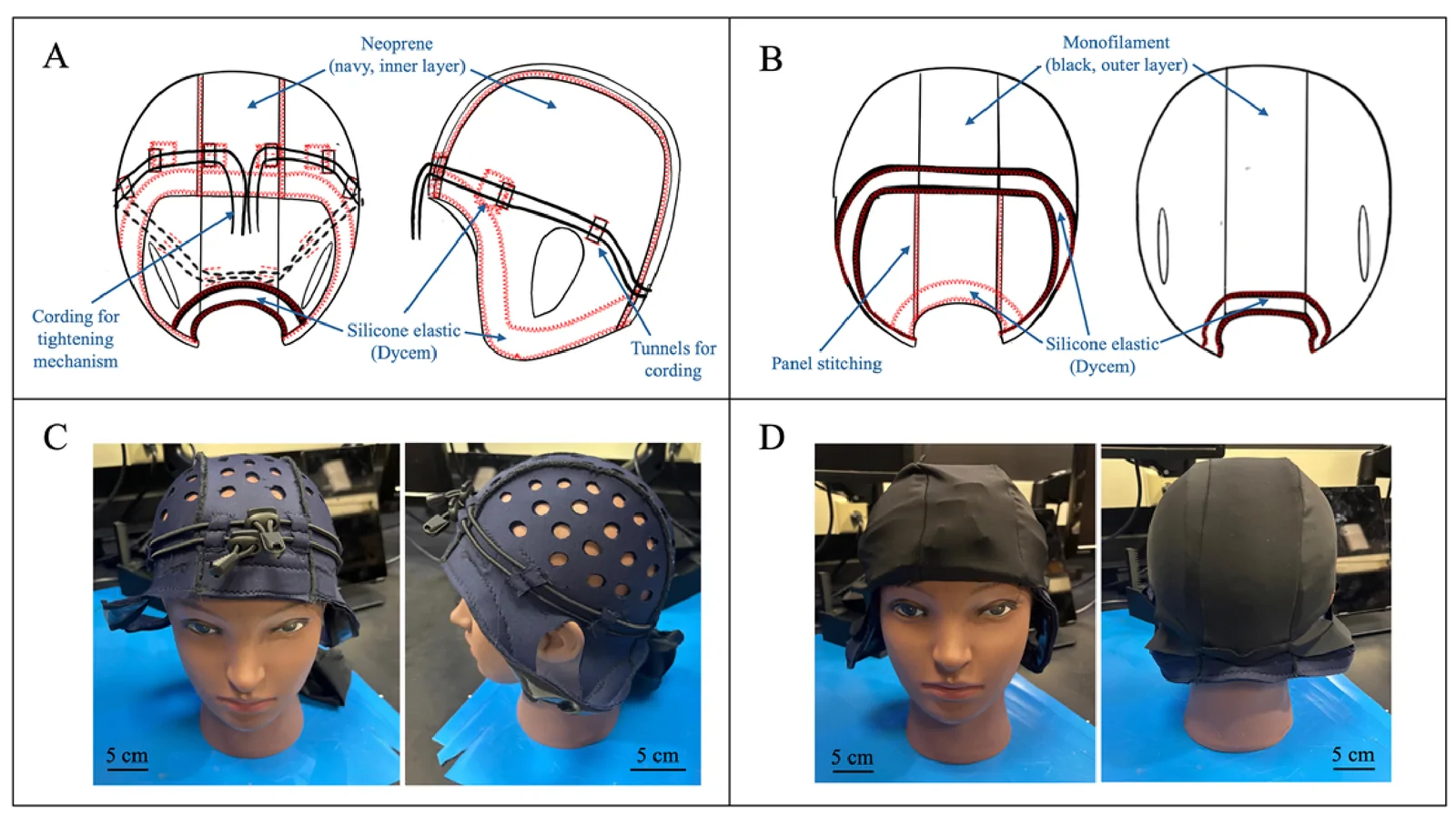
(a) Detailed fabric and sewing designs for the inner fabric layer and tightening mechanism and (b) for the outer layer of the cap. (c) Finalized images of the inner layer on a mannequin and (d) with the outer layer in place.

Our non-traditional tightening mechanism sits atop the inner layer and eliminates the need for a chinstrap, which is often the primary mechanism to secure a cap to a participant. The mechanism comprises a set of two elastic cords with a cord lock that runs around the circumference of the cap, as shown in Fig. 1(a) and Fig. 1(c). The mechanism ensures a secure fit, enhancing the ability of the Dycem to maintain contact with the scalp of the wearer. The design accommodates the entire head circumference and conforms to the lower back of the head, removing the need for a chinstrap.

The outer layer (Fig. 1(b)) is made of conformal neoprene fabric to secure the fNIRS system components, including optodes and system controllers, and to contain any protruding wires from fNIRS electronics or the tightening cords connected to the inner layer, preventing them from exiting the cap. By minimizing protrusion of these elements, the layer reduces the risk of cable displacement, entanglement with the surgical bed or chair, interference with surgical personnel or equipment, and motion-related signal artifacts.

### 2.2. Cap fabrication

To create the inner layer, we cut the neoprene fabric into three panels, referred to as the left side, right side and middle panels, according to measurements outlined in the “Patterning” design files available on the OSF page. Once cut, the middle panel was overlocked to the side panels with a 0.25-inch seam allowance. Then, we cut the Dycem into a one-inch strip matching the sizing provided in the “Patterning” design files and used a zigzag stitch to attach it to the rim of the inner layer. We then cut four squares (one inch by one inch) of the Dycem and attached them via zigzag stitch to the forward-facing side of the neoprene spanning the middle and both side panels. Eight strips of monofilament fabric into one-half inch by one-inch rectangles were cut to use as cording guides for the tightening mechanism. These strips were attached to the inner layer via straight stitches and spanned the middle and both side panels. Once this step was complete, we cut two pieces of cording at least five inches longer than the circumference of the inner layer, which varies depending on the desired size (XS – XL). We threaded both pieces of cording through all of the monofilament rectangles and added end caps and cord locks to finalize the tightening mechanism.

To create the outer layer, we cut the monofilament fabric into three panels and overlocked the panels with a 0.25-inch seam allowance, similar to the process that was described for the inner layer. Once we overlocked the cap, we then flipped its orientation, so the seam allowance faced outwards and lined the bottom edges to the inner layer. We then attached the outer layer to the inner layer at the aligned bottom edge using a zigzag stitch.

Our cap design offers broad adaptability across multiple cap-based fNIRS systems. This versatility is achieved by creating holes in the fabric that match the diameter of the intended fNIRS system’s optodes. Prior to deployment, the exact size of the intended optodes should be measured, and corresponding holes can be punched into the cap material using a dedicated punch tool matched to the diameter of the fNIRS system sensors; for this study, we used a tool provided by Artinis with the Brite system. This approach enables seamless integration of optodes while maintaining fabric integrity, allowing the same cap design to be used with a variety of fNIRS platforms.

To accommodate different imaging layouts while maintaining anatomical positioning, optode openings were placed in the custom cap in accordance with the international 10-10 system [14]. The use of standardized locations facilitates consistency across probe configurations and is compatible with landmark-based layout design tools such as fOLD [25]. This positioning strategy is also consistent with that of most commercially available fNIRS caps including the Artinis cap used as the control in this study. Researchers can implement a desired probe configuration by populating the predefined optode locations corresponding to the anatomical region and source-detector arrangement of interest, while unused optode locations remain unpopulated. This strategy allows alternative probe configurations to be implemented in standardized optode locations without requiring researchers to manually determine anatomical positions during cap preparation.

The current cap design allows majority of coverage across the international 10-10 system, but access to a subset of these predefined locations is limited by the tightening mechanism. The greatest restriction occurs in the anterior portion of the cap where the prefrontal (Fp) and all anterior frontal (AF) except AFz are obstructed. The lateral frontal locations F7-F10 are also unavailable; however, Fz and F1-F6 remain accessible for frontal coverage. The cap retains access to all fronto-central (FC), central (C), centro-parietal (CP), and parieto-occipital (PO) locations. The majority of temporal and parietal locations are also available, with restriction primarily affecting the most lateral positions (FT9-FT10, T9-T10, and P9-P10). In the occipital region O1 and O2 remain accessible, while Oz is obstructed. Therefore, although the tightening mechanism limits layouts requiring prefrontal or specific peripheral 10–10 locations, it does not prevent measurements across the broader frontal, temporal, parietal, and occipital regions.

To enable ready-access to the cap for researchers utilizing cw-fNIRS, the files associated with cap creation are available on our lab OSF page. The organization of the webpage and descriptions of the included design files associated with the cap are also annotated in the Supplement 1.

### 2.3. Experimental design

We recruited n = 30 participants (average age = 33.4 years, 13M/17F) to evaluate our custom cap. The first 23 participants tested the custom cap; this sample size was determined prior to the study based on pilot data and a preliminary power analysis in G*Power (v3.1) [26,27] designed to yield a power of at least 0.8 (i.e., 1 - β > 0.8), which is a generally accepted statistical threshold [28]. The latter seven participants tested a commercially available cap (Artinis Brite), which served as a control cap for comparison. The cap sizes were selected individually for each participant based on fit for testing both the custom (fabricated in five sizes XS, S, M, L, XL) and commercial caps. The sizes of the custom and commercial cap participant groups are not equal, as the comparison between groups was intended to be exploratory and non-powered, and the use of a smaller commercial group improved ease of recruitment.

The experimental procedure was approved by the Vanderbilt University Institutional Review Board (IRB#120). All methods were carried out in accordance with the relevant guidelines and regulations, and informed consent was obtained from all participants.

The procedure was designed to evaluate the cap across three areas: (1) resistance to motion, (2) ability to produce high-quality hemodynamic data, and (3) comfort for the participant. The main focus of the study was to compare the motion resistance, achieved signal quality, and comfort of the custom cap to acceptable thresholds within the fNIRS field. The experimental procedure was two hours in total length and followed the paradigm outlined in Fig. 2.

**Fig. 2.**
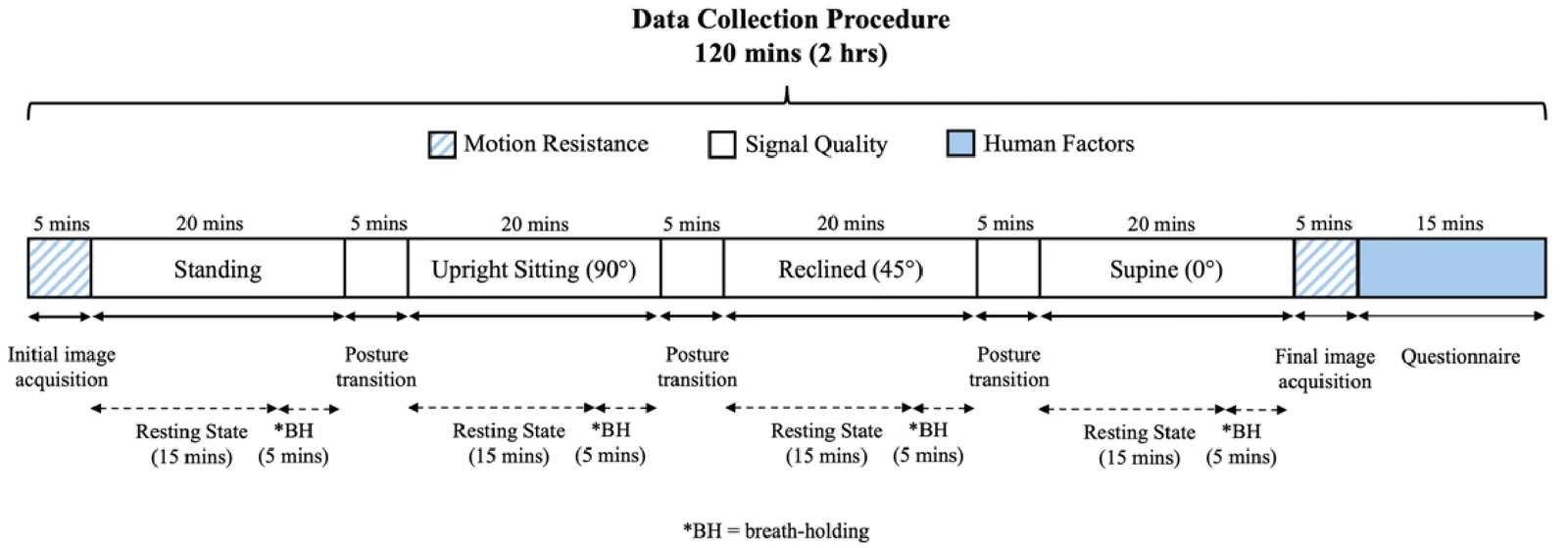
Breakdown of the experimental procedure timing including imaging acquisition for motion testing (blue crosshatch), postural settings and transitions for hemodynamic data collection and signal quality assessment (white), and human factors questionnaire (blue).

The procedure was divided into four 20-minute blocks designed to mimic aspects of the perioperative workflow and was performed in the mock operating room environment at VISE. The first block required the participant to stand within the mock operating room for 20 minutes to simulate a condition with no external support and greater freedom of motion that may occur before or after surgery but still within the perioperative window. The second block consisted of 20 minutes of upright sitting to mimic an alert state that would likely occur preoperatively. The third block was 20 minutes of reclined sitting to most closely mock a postoperative resting state. The final 20-minute block required the participant to assume a totally supine position, as would be done intraoperatively. Each block was separated by a five-minute transition period during which the participant prepared for and moved into the upcoming posture. These transition blocks simulated timing needed for the patient to transit to different postural states (e.g., to move to the surgical table). Because movements between postures also occur during the perioperative window, the cap was not adjusted or reset during these times.

#### 2.3.1. Assessment of cap motion resistance

The process used to test for resistance to motion was adapted from previous work by Wellborn et al. [23]; the modifications we implemented were chosen to more closely reflect cardiac surgery workflows. Motion resistance was assessed by tracking displacement of the cap relative to the participant’s head using visual markers and standardized imaging. To enable this, the cap was marked prior to testing with a fabric marker to create landmarks for motion tracking.

Prior to image acquisition and testing, the cap was populated with the electronics of a commercial fNIRS system (Artinis Medical Systems – Gelderland, The Netherlands) and positioned atop the head of the participant. The researchers then used a wooden stick that accompanied the Artinis fNIRS system and worked to the best of their ability to move hair strands out of the way of each optode. Once each participant was prepared and wearing the cap, we collected four initial images, as shown in Fig. 3, and the participant was instructed to assume the standing posture used to start hemodynamic and motion testing. Images were collected from standardized locations around the head using a Samsung Galaxy A7 Lite tablet to register cap position and assess movement over time.

**Fig. 3.**
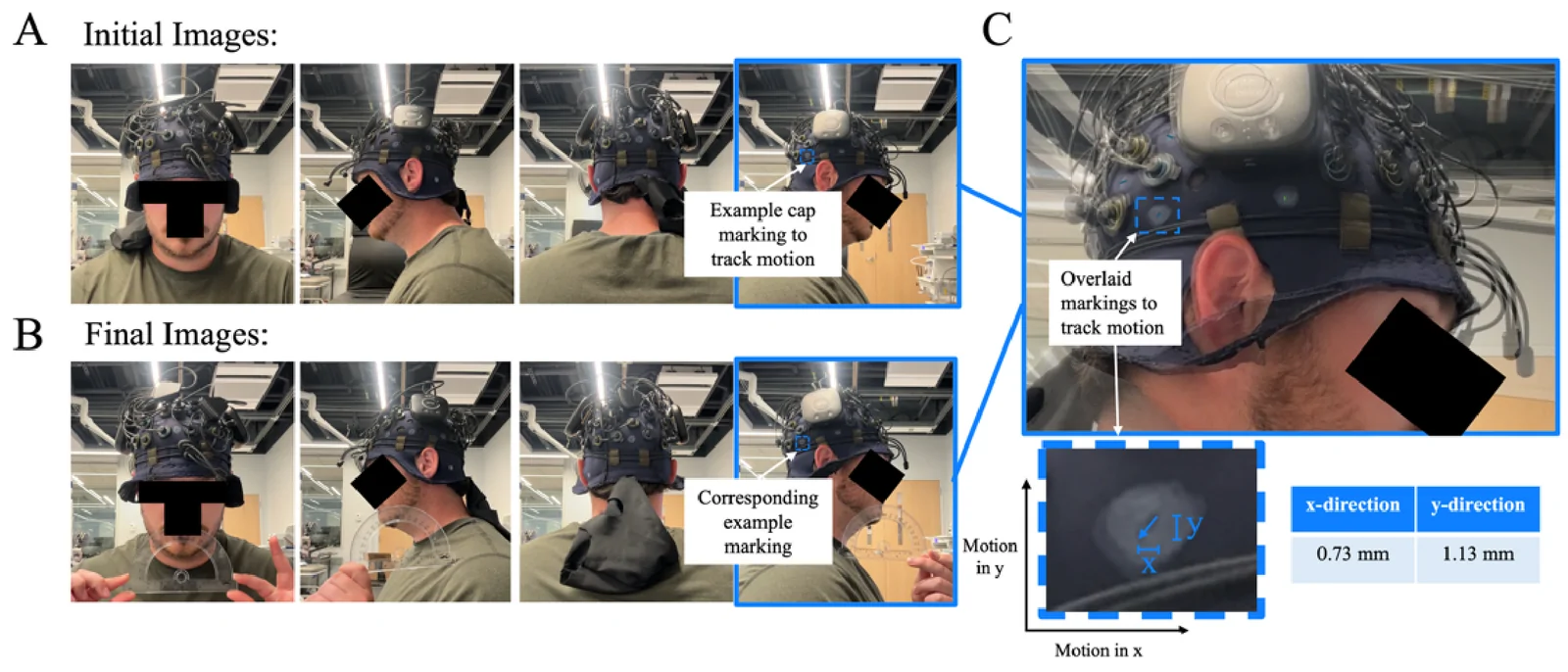
Overview of data collected during motion resistance testing. From the set of four images taken in standard positions (a) before and (b) after the procedure, corresponding images are (c) overlaid to determine the amount of cap movement in the x- and y-directions. Cap is shown here with the outer layer removed to visualize alignment markings.

Once all posture blocks were completed, four final images were captured from the same standardized locations in relation to the participant and with the same tablet camera. The four initial and final images were then overlaid using FIJI, which is an open-source extension of ImageJ used widely in biomedical image analysis [29]. FIJI provides a framework with numerous plugins for feature detection, tracking, and spatial registration. For this study, cap motion was quantified by tracking defined visual landmarks across time using FIJI’s multi-point and tracking tools, and inter-frame registration was performed using the BigWarp plugin, which enables interactive landmark-based alignment and deformation analysis between image frames. BigWarp was used to compute spatial transformations between frames, allowing quantification of translational displacement of the cap relative to the head between the acquired before and after images.

Within FIJI, the cap landmarks were used to calculate the amount of cap movement across the entire procedure. This translational displacement was captured in the x- (lateral movement) and y-directions (vertical movement) relative to the heads of each participant. For the front- and back-facing images, these coordinate axes were defined directly from the registered image plane with the x-axis corresponding to horizontal displacement and the y-axis corresponding to vertical displacement. For the left- and right-facing images, the coordinate system was reoriented to align with anatomical landmarks such that the x-axis followed the line from the center of the eyes to the superior auricular point of the ears and the orthogonal direction represented the y-axis or vertical displacement. The displacement in each direction was compared to a threshold of 5.28 mm of allowable error. This error threshold was calculated using data from the multimodal Human Connectome Project (HCP) atlas [30]. Because the cap is intended for general-purpose use across cortical regions, nearest-neighbor distances were computed for all cortical parcels by measuring each parcel’s minimum surface distance to an adjacent parcel along the cortical mesh. This calculation produced a mean nearest neighbor surface distance of approximately 10.56 mm. Using half-distance (Nyquist sampling limits) to avoid crossing into neighboring parcels, we arrived at the threshold of 5.28 mm.

Once data were collected from the participants, they were averaged in the x- and y-directions and t-tests were conducted to compare the means to the constant threshold. The testing against the constant threshold was performed for both the custom and commercial caps, and exploratory testing, also using t-tests, was also performed to compare between the cap groups.

#### 2.3.2. Signal processing and quality evaluation

The hemodynamic data from the procedure were collected with the Oxysoft software (v3.4.12) used to operate the Artinis Brite system. To evaluate signal quality, raw light intensity data were converted to optical density using the Homer3 toolbox in Matlab [31]. These processed optical density signals were then used to compute signal quality metrics and evaluate hemodynamic responses.

To ensure the cap enables high quality signal acquisition, we collected data throughout all four 20-minute blocks and through each posture transition period. The data were post-processed and assessed via signal-to-noise ratio (SNR) and scalp-coupling index (SCI), two common metrics in fNIRS signal quality assessment [28,32]. SNR and SCI were computed from the preprocessed optical density signal using custom Matlab scripts implemented in Homer3.

Channels were pruned from analysis based on the signal quality from the first baseline period (first 15 minutes of data collection during standing posture). Data with SNR < 5 dB or negative SCI over this time frame were removed from analysis due to issues with cap setup (e.g., improper scalp coupling to begin the procedure or insufficient signal quality through hair).

Higher signal quality in fNIRS is usually recognized as increased SNR (such as > 10 dB) and SCI (> 0.8) [28,32,33]; however, these thresholds are not universally defined and are not required to obtain meaningful signal, as channels below these values can still yield reliable hemodynamic responses [34]. Therefore, we compared the averaged SNR and SCI values for both the custom and commercial cap groups and report the proportion of channels that exceeded these standard values.

To further verify signal quality, the final five minutes from each block were utilized to perform a breath-holding (BH) procedure. Breath-holding procedures are commonly used in fNIRS assessments to demonstrate a robust hemodynamic response and functionally verify signal integrity [35,36]. We utilized the task to confirm that we could reliably capture the characteristic changes in the hemoglobin species. Within the five-minute procedure, participants performed breath-holding across four 20-second trials. Hemodynamic responses were computed by converting the bandpass filtered (0-0.2 Hz) optical density signal to changes in oxy- and deoxy-hemoglobin using the Modified Beer-Lambert law, and responses were averaged across trials for each posture block and each participant.

#### 2.3.3. Human factors and user comfort assessment

To assess and compare the comfort and usability of the commercial fNIRS cap and the custom lab-designed cap, we conducted a human factors evaluation using a structured comfortability questionnaire. The questionnaire consisted of 18 statements, each rated on a 1–10 Likert scale, where one indicated strong disagreement and 10 indicated strong agreement. Because comfort and fit are subjective and often involve subtle perceptual differences, a 10-point Likert scale was selected to allow participants to express fine gradations in comfort that may not be captured by coarser five-point scales. The questions targeted multiple domains of user experience, including initial and sustained comfort, pressure distribution, sensor-induced discomfort, thermal regulation, ease of adjustment, mental fatigue, and overall willingness to use or recommend the cap. An optional open-ended section followed the scaled questions, allowing participants to report specific discomforts, pressure points, or suggestions for design improvement. The full set of questions is provided in the Supplement 1.

Our analysis of the questionnaire results focused on investigating whether participant responses were generally positive. Because questionnaire response data are discrete, we conducted nonparametric testing using the one-sample Wilcoxon signed-rank test to evaluate whether the mean rating differed significantly from the midpoint of the scale (a value of five). This approach allowed us to determine whether ratings were significantly more positive than neutral.

We also performed an exploratory comparison between the commercial cap and our custom cap. Because responses were collected from two independent groups, Mann-Whitney testing was used to compare means scores between groups. Also, given the exploratory nature of the analysis, multiple comparisons were not formally corrected.

## 3. Results

A breakdown of the participant demographics for the study is included in Table 1. The study included 30 participants spanning 18–60 years of age, representing male and female participants and multiple self-reported racial and ethnic groups. The custom cap was manufactured in five sizes (XS–XL), with cap size selected individually for each participant; therefore, the cap was evaluated across a variety of participant demographics, while the availability of multiple cap sizes was designed to accommodate differences in head size. Specific hair type and hairstyle characteristics were not systematically recorded.

**Table 1.** Participant Demographics from Study

Characteristic	Overall Sample (n = 30)	Custom Cap (n = 23)	Commercial Cap (n = 7)
Age, mean (range), years	33.4 (18-60)	36.1 (18-60)	24.4 (20-32)
Gender, quantity			
Female	17 (57%)	15 (65%)	2 (29%)
Male	13 (43%)	8 (35%)	5 (71%)
Prefer not to answer	0 (0%)	0 (0%)	0 (0%)
Race/ethnicity^*a*^, quantity			
White	20 (67%)	17 (74%)	3 (43%)
Asian	7 (23%)	4 (17%)	3 (43%)
Black or African American	2 (7%)	1 (4%)	1 (14%)
Hispanic or Latino	2 (7%)	2 (9%)	0 (0%)
Not Hispanic or Latino	3 (10%)	1 (4%)	2 (29%)
American Indian or Alaska Native	0 (0%)	0 (0%)	0 (0%)
Native Hawaiian or Other Pacific Islander	0 (0%)	0 (0%)	0 (0%)
Prefer not to answer	0 (0%)	0 (0%)	0 (0%)

^*a*^ Participants could select all categories that applied; therefore, race/ethnicity categories are not mutually exclusive, and percentages do not necessarily sum to 100%.

### 3.1. Assessment of cap motion resistance

#### 3.1.1. Custom cap

Figure 4 reports the motion data associated with translational displacement of the cap in the x- (horizontal) and y- (vertical) directions across the full experimental procedure, as measured from the cap fiducial markers tracked between initial and final images for each participant (n = 23). Displacement was calculated relative to the head of each participant using a head-centered coordinate frame that was established via image registration and discussed previously in Section 2.3.1.

**Fig. 4.**
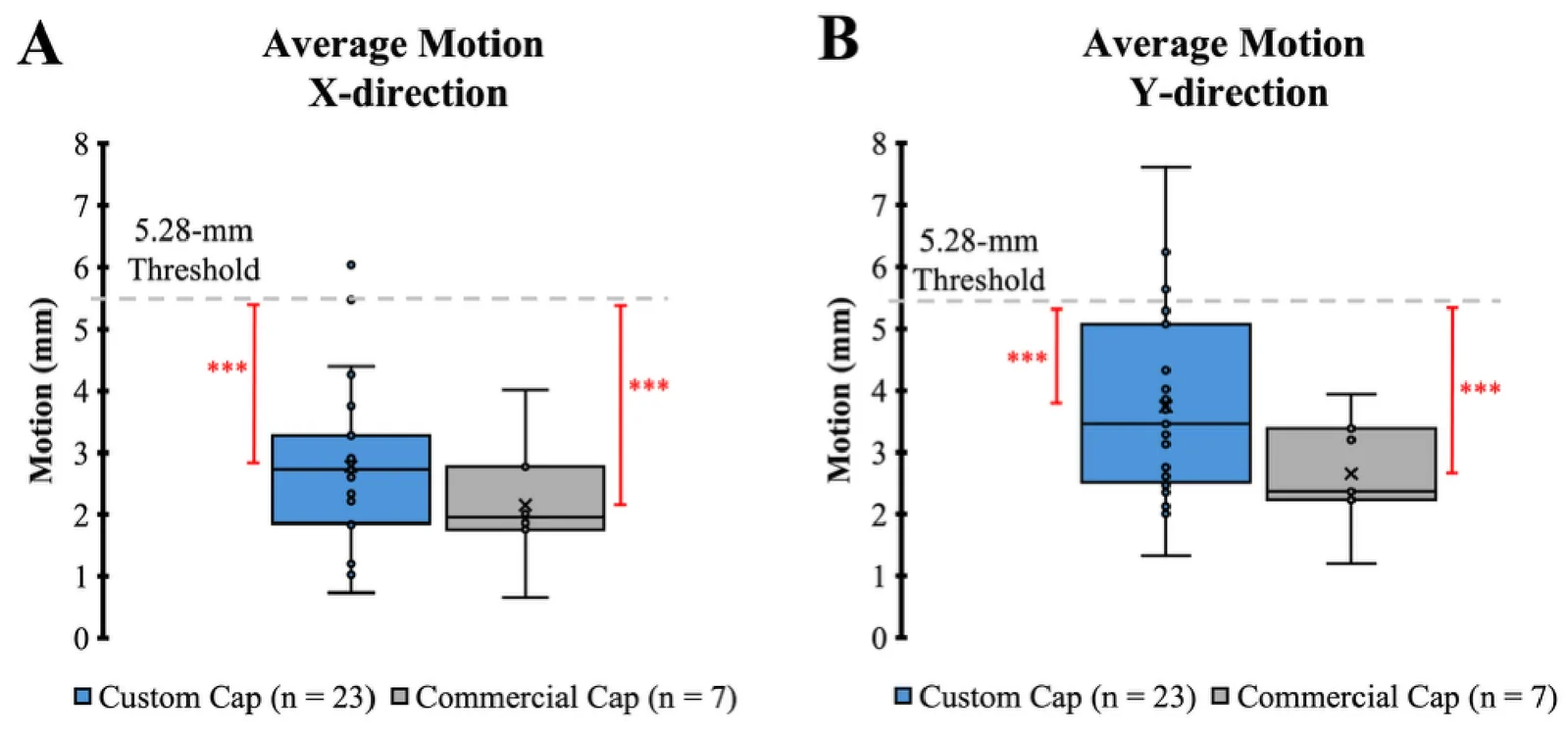
Results from motion resistance testing in the (a) x- and (b) y-directions for both the custom and commercial Brite cap systems. Comparisons between groups in both the x- and y-directions were conducted but no statistically significant result occurred.

In the x-direction, the average motion over the procedure was 2.78 mm (SD = 1.33 mm), which was well below the predefined 5.28-mm threshold (Fig. 4(a)). In the y-direction, the average motion was 3.74 mm (SD = 1.53 mm), which also fell below the 5.28-mm threshold (Fig. 4(b)).

The motion data were tested for normality using skewness and kurtosis as the primary metrics. The calculated skewness (0.812 in the x-direction, 0.779 in the y-direction) and kurtosis (0.739 in x, 0.393 in y) values were within acceptable ranges for parametric analysis [37]. A one-sample t-test confirmed that motion in both directions was significantly less than the predefined threshold of 5.28 mm (p = 3.75 × 10^−9^ in x; p = 3.95 × 10^−5^ in y), with post hoc power analysis indicating excellent statistical power (1.00 in x; 0.999 in y) compared to the generally accepted threshold of 0.8 (i.e., 1 - β > 0.8) [28].

Compared to horizontal motion, there was increased vertical shifting, which likely stems from the novel tightening mechanism and lack of chinstrap. In the absence of the typical, robust vertical restraint of the chinstrap, small upward or downward shifts are more likely to occur due to gravity, facial movement, or minor postural adjustments during the procedure.

Nevertheless, motion remained below the 5.28-mm threshold in both axes, with particularly robust performance in the horizontal direction. The comparatively higher variability in vertical shifting suggests a potential avenue for future design refinement (e.g., modifying the circumferential tightening mechanism or increasing the use of Dycem) to further reduce vertical displacement and increase consistency for use in OR environments.

#### 3.1.2. Commercial cap

For the commercial cap (n = 7), average motion was 2.28 mm (SD = 1.03 mm) in the x-direction and 2.82 mm (SD = 0.913 mm) in the y-direction. One-sample t-tests in both directions showed that the means were significantly below the 5.28-mm threshold (p = 1.28 × 10^−4^ in x; p = 1.90 × 10^−4^ in y).

#### 3.1.3. Cap comparison

In comparing the two cap designs, the commercial cap averaged less motion in both the x- and y-directions. The difference was largest in the y-direction, where the custom cap shifted nearly 1 mm more per participant. As described previously, this additional vertical motion from the custom cap is likely influenced by the lack of chinstrap and suggests that future iteration to the novel tightening mechanism may help to improve motion resistance.

As an exploratory comparison, we applied two-sample independent t-tests in the x- and y-directions to determine if the means across the two caps differed from each other. No statistically significant difference was observed in either direction (p = 0.288 in x and p = 0.0524 in y). Although the smaller sample size for the commercial cap limits statistical power, these results suggest that the custom cap performs comparably to the commercial system while maintaining motion within acceptable limits.

### 3.2. Signal processing and quality evaluation

#### 3.2.1. Custom cap

SNR values, averaged across channels within each participant and then across participants (n = 23), yielded a mean SNR of 21.1 dB (SD = 3.87), which is well above what is commonly considered higher signal quality (>10 dB) (Fig. 5(a)). Further, across the full dataset, 97.2% of retained channels achieved SNR > 10 dB over the full procedure demonstrating robust signal fidelity across extended monitoring durations in a mock OR environment.

**Fig. 5.**
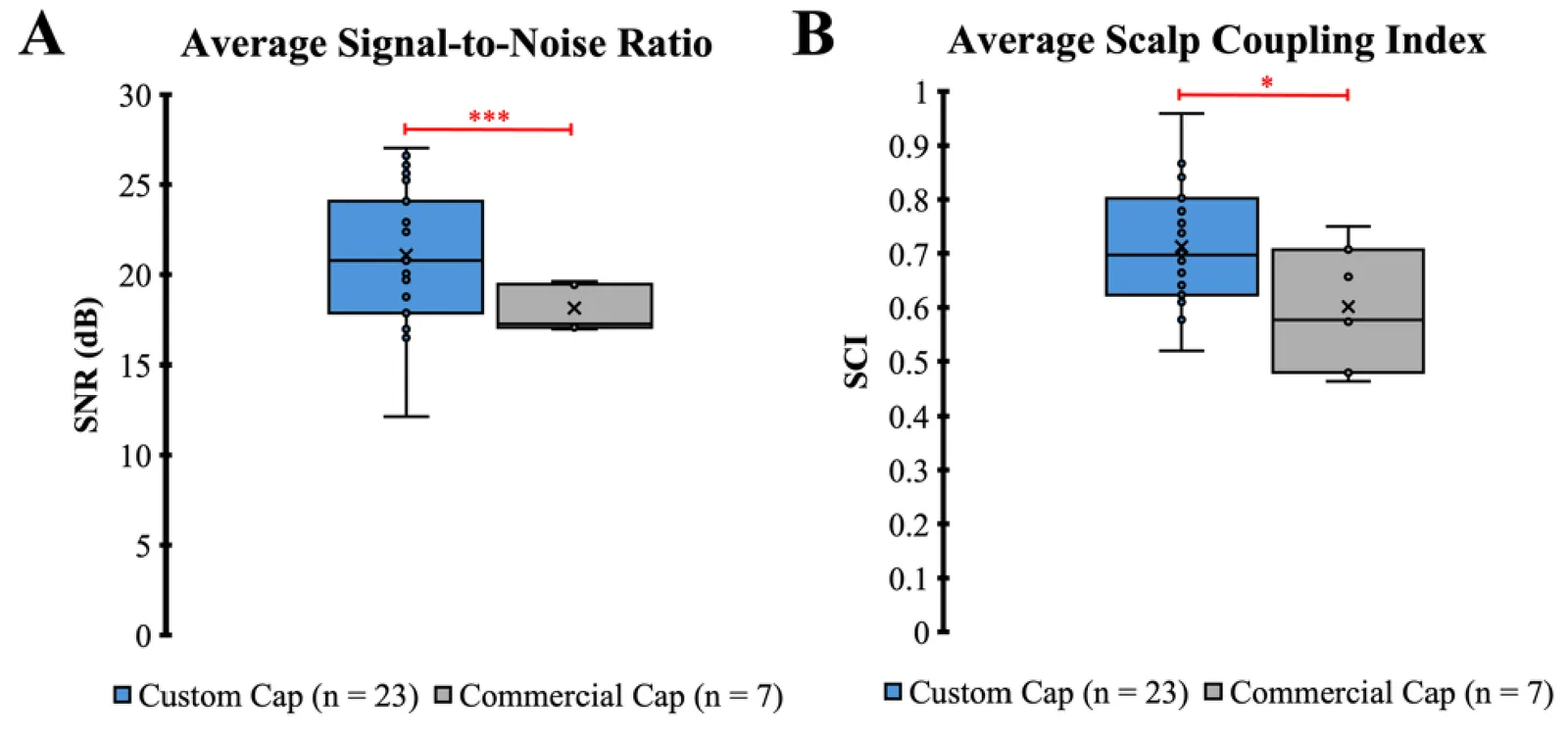
Results from signal quality testing through (a) SNR and (b) SCI metrics. Significant differences in both SNR and SCI were found between the custom and commercial caps, but these likely resulted from the smaller sample size and more diverse participant demographics of the commercial cap group.

The mean SCI across participants was 0.713 (SD = 0.107) (Fig. 5(b)), and over half of the retained channels (53.1%) produced SCI above 0.8 for the procedure, which is considered higher signal quality in fNIRS.

Taken together, these results demonstrate that the custom cap provides consistently high signal quality across data collection sessions. The high SNR values observed across nearly all channels indicate robust signal quality, while SCI values suggest generally strong scalp-to-optode coupling with a substantial proportion of channels achieving higher-quality thresholds. The observed variability in SCI and overall average falling below 0.8 is likely influenced by participant-specific factors such as hair characteristics and scalp morphology rather than instability of the cap itself. Importantly, these signal quality measures were obtained under prolonged and varied postural conditions and in a mock OR environment, supporting reliable data acquisition with the cap design in such settings.

#### 3.2.2. Commercial cap

For the commercial cap (n = 7), the mean SNR was 18.1 dB (SD = 1.28), and 96.9% of retained channels achieved SNR > 10 dB. While the SNR data demonstrates strong quality, the mean SCI across participants was 0.601 (SD = 0.109) and only 29.2% of retained channels produced SCI above 0.8 for the procedure.

#### 3.2.3. Cap comparison

The proportion of channels retained for this analysis were comparable between cap conditions. Based on the predefined signal-quality criteria (SNR < 5 dB or negative SCI over the first 15 minutes of data collection), 46.3% of channels were pruned for the custom cap and 46.4% for the commercial cap. The custom cap produced higher averages for SNR (∼3 dB higher) and SCI (∼0.11 higher) and a greater proportion of channels achieving higher quality (∼24% higher) than the commercial cap. Exploratory comparisons were conducted using two-sample independent t-tests for SNR and SCI across the two caps. For each metric, a statistically significant difference was observed (p = 3.24 × 10^−^3 for SNR and p = 0.0293 for SCI).

The difference in these signal quality results between caps should be interpreted with caution given the smaller sample size for the commercial cap and the increased proportion of diverse participants in comparison to the commercial cap (Table 1): the majority of participants that used the custom cap self-identified as White, whereas less than half of participants that used the commercial cap self-identified as White (three of seven). This disparity in the diversity demographic stems from our random recruiting process, as we did not explicitly control for ethnicity during recruitment. We hypothesize that the lower SNR and SCI resulting from the commercial cap stem from factors that cause signal loss in diverse individuals (e.g., hair color and thickness, scalp size and shape) [38] and not from any differences in design implemented in our custom cap.

In addition to analyzing the produced signal quality across the entire data collection duration, we also considered the data across the discrete posture intervals to determine if cap performance varied across posture. Across the 20-minute blocks of standing, upright sitting, reclined, sitting, and supine, the signal quality did vary (Table ); however, there was no consistent posture-dependent pattern for either cap. Specifically, the posture-wise fluctuations in signal quality did not indicate a consistent reduction in performance of the custom cap relative to the commercial cap in any posture.

#### 3.2.4. Representative breath-holding responses

In order to investigate the cap performance associated with hemodynamic signals, we examined the task-induced breath-holding responses. The breath-holding data were first processed and block-averaged across all posture conditions for each participant to assess signal quality across the entire duration of the extended monitoring session. The participant-wise data were then group averaged to represent the custom (n = 23) and commercial (n = 7) cap groups.

As shown in Fig. 6, the group-averaged breath-holding data shows the expected rise in deoxygenated-hemoglobin and subsequent drop in oxygenated-hemoglobin during the breath-holding period (shaded gray) before inverting during recovery [36]. This response pattern was consistently observed during the initial participant-wise data both across participants and across the different caps.

**Fig. 6.**
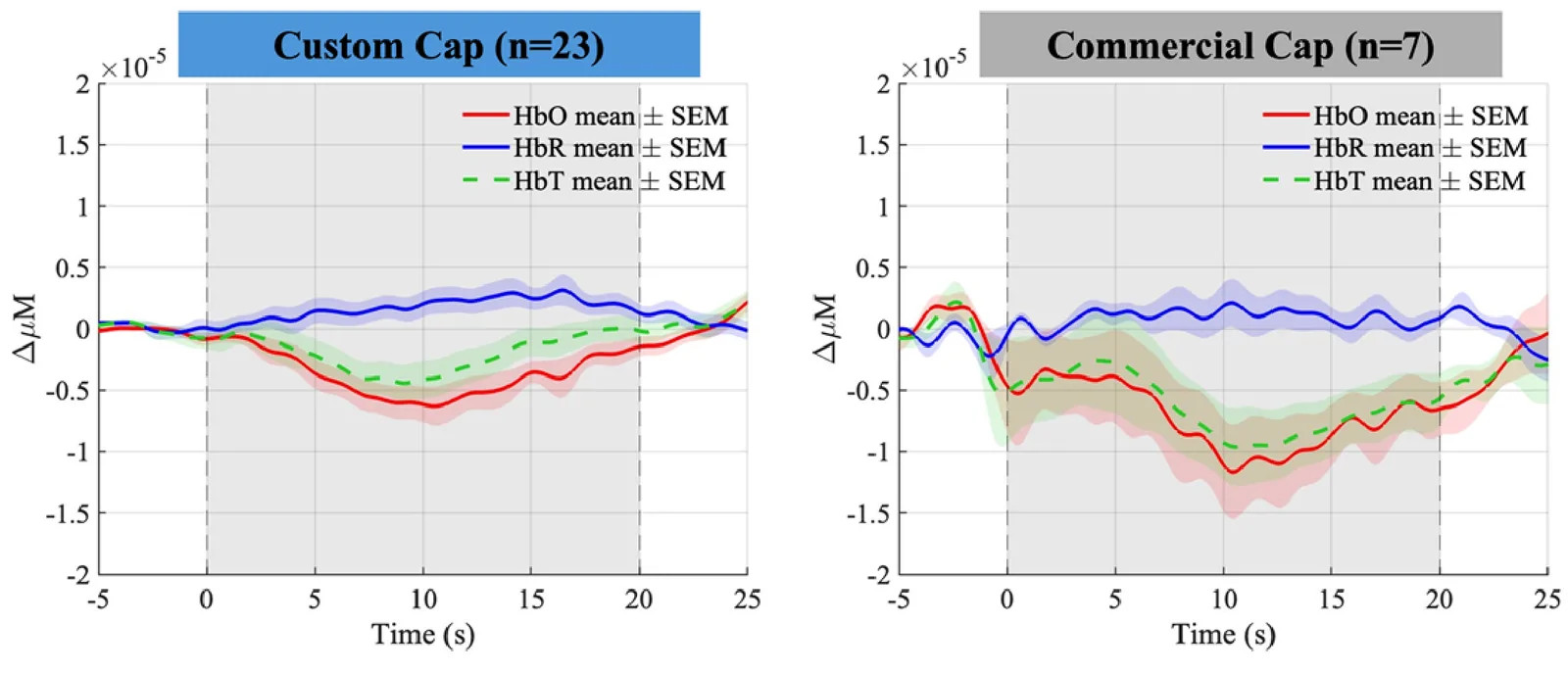
Block-averaged and group-averaged breath-holding responses from the custom and commercial cap groups. The custom cap captures the expected rise in deoxy-Hb and subsequent fall in oxy-Hb similar to the commercial cap.

In addition to evaluating the hemodynamic responses qualitatively, we calculated the Pearson correlation coefficients for the HbO and HbR time series across the custom and commercial cap group to quantify similarity. The correlation coefficients (*r*) were calculated over the full analysis window plotted (-5 to 25 seconds). The HbO time series demonstrated strong positive correlation between groups (r = 0.849), and the HbR time series demonstrated moderate positive correlation (r = 0.633).

Although the breath-holding data demonstrated the expected increase in HbR and decrease in HbO, total hemoglobin concentration (HbT) decreased during breath holding. HbT is generally expected to increase due to increased cerebral blood volume associated with hypercapnia [39,40]. We hypothesize the decrease in HbT resulted from the instruction given to participants around the breath-holding periods; unlike in other work, we did not explicitly ask participants to perform the breath hold at the end of expiration [35,36,39,40].

While the decrease in HbT limits interpretation of the data as a canonical hemodynamic response to breath holding, this decrease was observed in both the custom and commercial cap groups. Further, the HbT time courses were moderately correlated (r = 0.593) indicating similar behavior between cap groups and suggesting that the unexpected HbT response was not specific to the custom cap design.

Collectively, these results support that the custom cap is capable of capturing physiologically meaningful hemodynamic signals during prolonged recordings and under varying postural conditions. Further, the ability to observe consistent responses across participants with varying demographic characteristics (e.g., gender and age) suggests that the cap performs reliably across differences in head geometry, while maintaining signal quality comparable to that obtained with a commercial system.

### 3.3. Human factors and user comfort assessment

#### 3.3.1. Cap comfort

Participants reported high levels of comfort with the custom cap across all comfort-related items (n = 23), with mean ratings significantly above the neutral midpoint of the scale (Fig. 7).

**Fig. 7.**
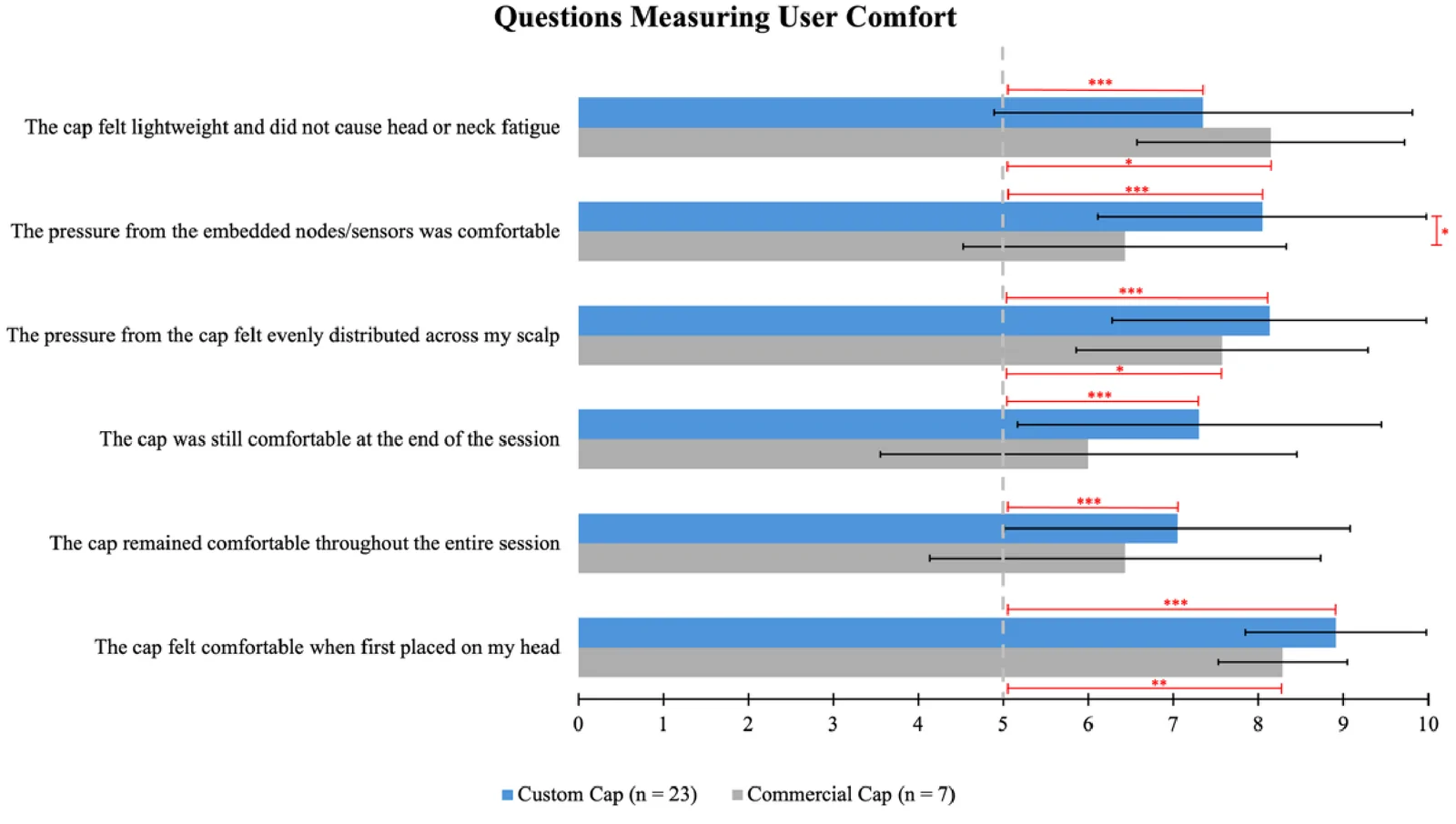
Results from the human factors survey where participant comfort was assessed.

Participants agreed that the cap felt lightweight and did not cause head or neck fatigue (M = 7.35, SD = 2.46, p = 3.90 × 10^−4^, β = 0.961), supporting its suitability for extended procedures.

Participants also reported that pressure from the embedded nodes/sensors was comfortable (M = 8.04, SD = 2.23, p = 6.83 × 10^−5^, β = 1.00) and that pressure from the cap was evenly distributed across the scalp (M = 8.13, SD = 1.96, p = 3.30 × 10^−5^, β = 1.00). These findings suggest that the cap’s mechanical design and load distribution were well tolerated and did not produce focal discomfort, which is an important consideration for extended wear.

Ratings related to sustained comfort over time were also positive, though comparatively more variable. Participants reported that the cap was still comfortable at the end of the session (M = 7.30, SD = 2.14, p = 4.15 × 10^−4^, β = 0.989) and remained comfortable throughout the entire session (M = 7.04, SD = 2.03, p = 3.48 × 10^−4^, β = 0.976). The greater variability across these data may reflect individual sensitivity differences caused by extended wear.

Initial placement comfort was rated strongly (M = 8.91, SD = 1.08), with significance (p = 1.18 × 10^−5^), per one-sample Wilcoxon signed-rank testing, and excellent statistical power (1.00).

Across the commercial cap, participants (n = 7) reported a similar, general feel for comfort with the cap, as all averaged responses to these comfort-related questions were above a value of five (Fig. 7). Among the responses that were deemed to be significantly above the threshold, participants agreed the cap was lightweight and did not cause head or neck fatigue (M = 8.14, SD = 1.57, p = 0.0156), that the pressure from the cap felt evenly distributed across the scalp (M = 7.57, SD = 1.72, p = 0.0156), and that the cap was comfortable when first placed on the head (M = 8.29, SD = 0.756, p = 7.81 × 10^−3^).

Mann-Whitney testing was used to compare responses between the two caps, and only one question returned a statistically significant relationship: participants rated the pressure from the embedded nodes/sensors as significantly more comfortable in the custom cap than with the commercial cap (p = 0.0358).

In pairing the isolated significant relationship with the fact that the custom cap averaged higher comfort ratings (though not significant) in five of the six comfortability evaluations, these findings suggest a strong overall perception of comfort for the custom cap and support the cap’s feasibility for prolonged intraoperative or clinical applications.

#### 3.3.2. Cap discomfort

For the custom cap (n = 23) ratings across all discomfort-related items were low and fell significantly below the neutral midpoint of the scale, indicating minimal perceived burden during use (Fig. 8). Participants strongly disagreed that the cap produced distracting sounds or sensations (M = 1.09, SD = 0.288, p = 1.98 × 10^−6^) with full statistical power (1.00). Reports of mental fatigue attributable to the cap were similarly low (M = 1.78, SD = 1.41, p = 1.32 × 10^−5^, β = 1.00), suggesting that the device did not impose meaningful cognitive or attentional burden during the procedure. Feelings of restriction or tension were also minimal (M = 2.35, SD = 1.56, p = 2.65 × 10^−5^, β = 1.00), further supporting the mechanical tolerability of the cap.

**Fig. 8.**
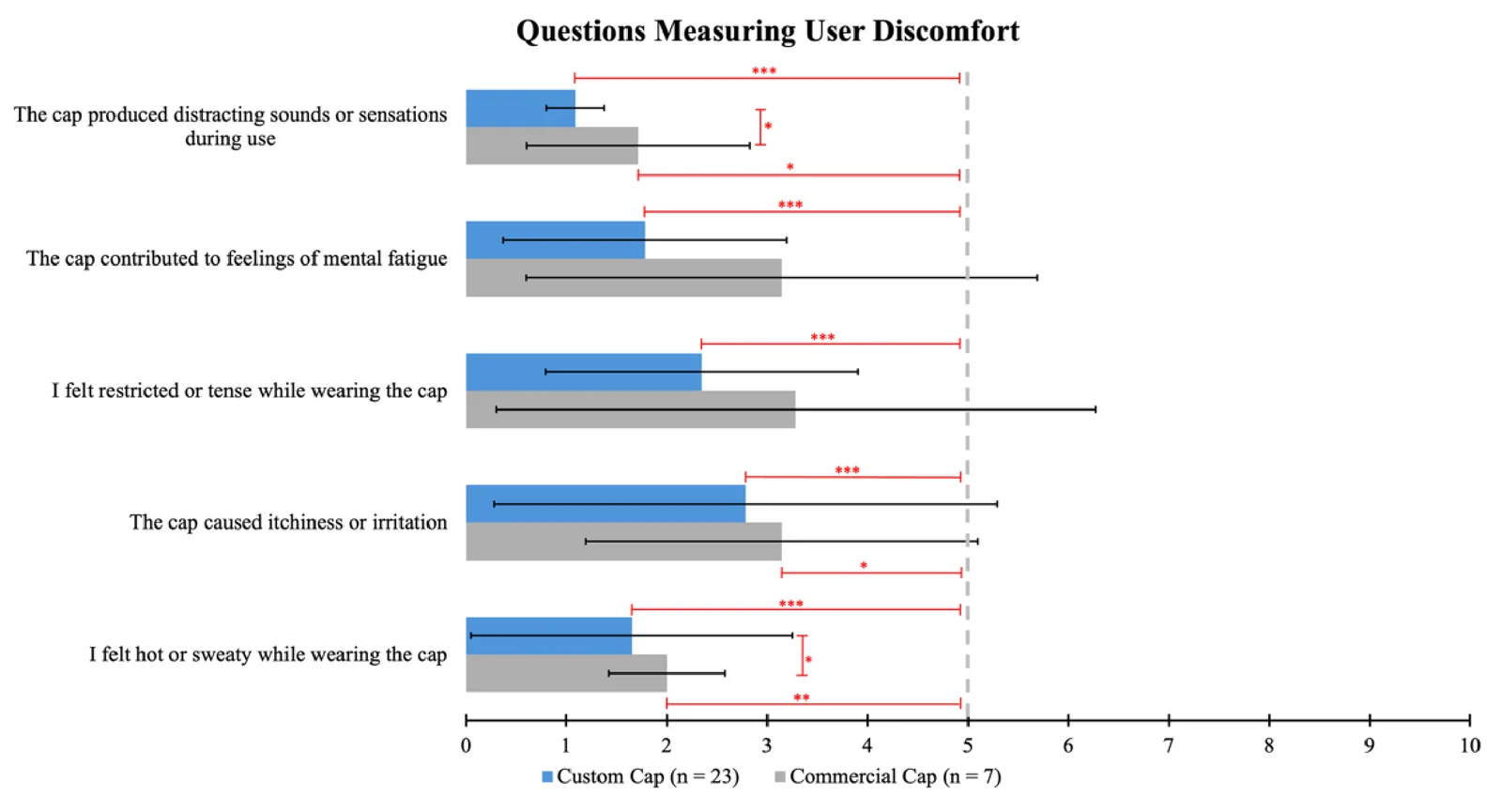
Results from the human factors survey where participant discomfort was assessed.

Skin and thermal discomfort were also limited. Reports of itchiness or irritation were significantly below neutral (M = 2.78, SD = 2.50, p = 7.30 × 10^−4^, β = 0.929), though variability was somewhat greater for this item compared to others. This dispersion likely reflects individual differences in scalp sensitivity, hair type, or skin reactivity rather than a limitation in the cap design. Participants also largely disagreed that they felt hot or sweaty while wearing the cap (M = 1.65, SD = 1.72, p = 3.57 × 10^−5^, β = 1.00).

For the commercial cap, participants (n = 7) again generally disagreed with the notation that the cap produced discomfort, as all averaged responses to these questions were below a value of five (Fig. 8). The responses that were deemed to be significantly below the threshold include that participants disagreed the cap produced distracting sounds or sensations (M = 1.71, SD = 1.11, p = 7.81 × 10^−3^), caused itchiness or irritation (M = 3.14, SD = 1.95, p = 0.0391), and that they felt hot or sweaty while wearing the cap (M = 2.00, SD = 0.577, p = 7.81 × 10^−3^).

Mann-Whitney testing produced two statistically significant relationships: participants rated the custom cap producing distracting sounds or sensations (p = 0.0215) and made them feel hot or sweaty (p = 0.0340) significantly lower than participants did for the commercial cap.

Again, similar to the analysis across comfort-related questions, the custom cap showed higher average ratings than the commercial cap across all five questions with two leading to significant differences. These discomfort data demonstrate a consistently low burden of the cap across thermal, cognitive, and sensory domains. This low reported discomfort, considered alongside the strong comfort ratings, supports the cap’s suitability for prolonged use in perioperative environments.

#### 3.3.3. Cap usability

For the custom cap (n = 23), participants reported very strong usability ratings related to setup, task interference, and overall acceptance, with all items scoring significantly above the neutral midpoint of the Likert scale (Fig. 9).

**Fig. 9.**
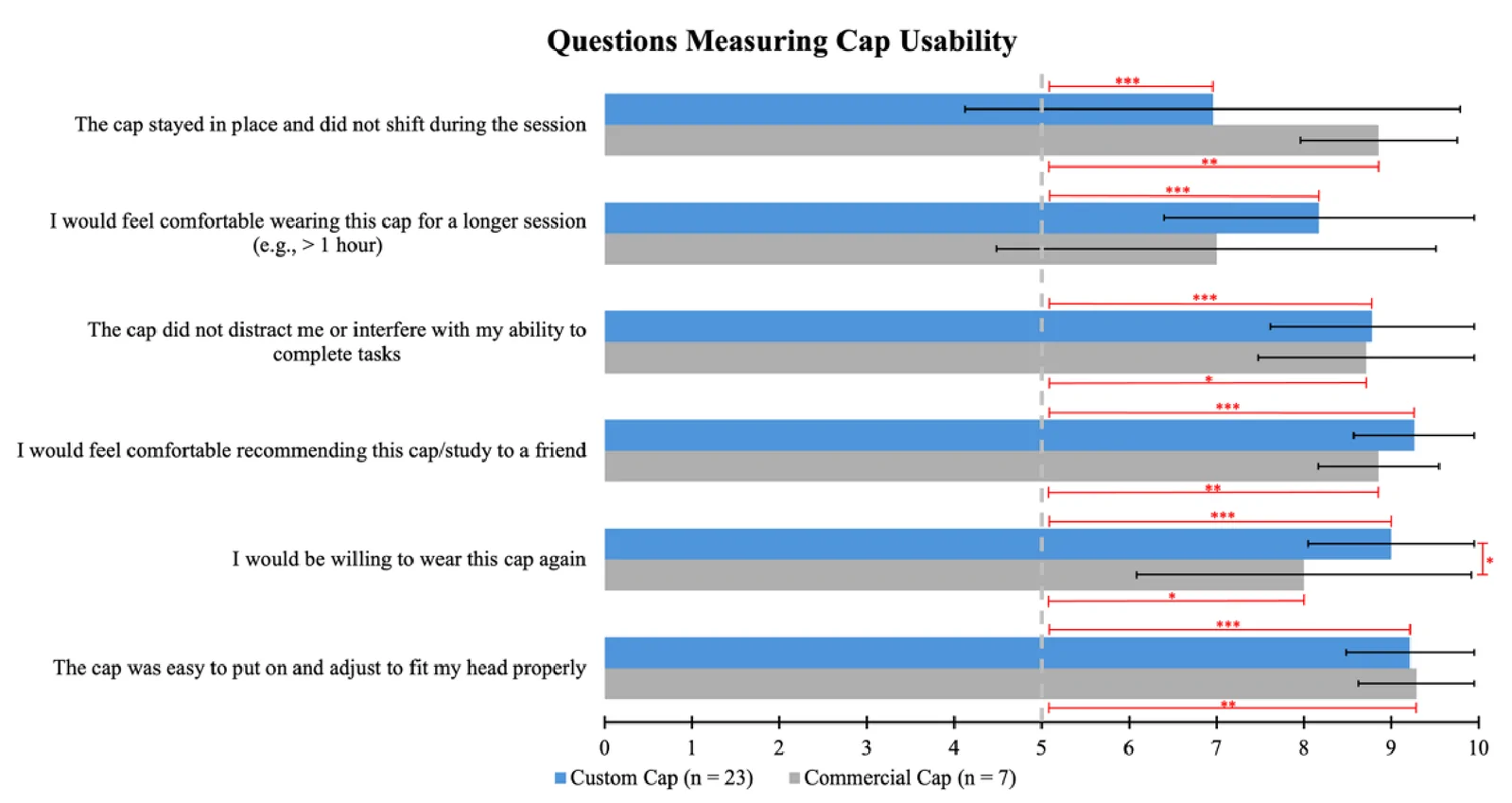
Results from the human factors survey where cap usability was assessed.

Participants agreed that the cap stayed in place and did not shift during the session (M = 6.96, SD = 2.84, p = 2.56 × 10^−3^, β = 0.731). Although this item demonstrated the greatest variability and lowest achieved statistical power, responses remained significantly above neutral and in the positive range. Importantly, even with this dispersion, the overall agreement that the cap remained stable aligns with the objective motion-resistance findings reported earlier, providing converging subjective and quantitative evidence of motion resistance.

Items directly related to extended use and positional stability, which were the central motivations for the development of the cap, demonstrated positive and statistically significant results. Participants agreed that they would feel comfortable wearing the cap for a longer session (>1 hour) (M = 8.17, SD = 2.39, p = 9.51 × 10^−5^, β = 1.00), indicating confidence in the cap’s suitability beyond the study duration.

Participants strongly agreed that the cap did not distract from or interfere with task completion (M = 8.78, SD = 1.35, p = 1.19 × 10^−5^, β = 1.00).

Willingness-based measures were similarly robust; participants indicated they would feel comfortable recommending the cap/study to a friend (M = 9.26, SD = 1.42, p = 8.70 × 10^−6^, β = 1.00) and would be willing to wear the cap again (M = 9.00, SD = 1.93, p = 1.73 × 10^−5^, β = 1.00)

Finally, the custom cap was rated as easy to put on and adjust (M = 9.22, SD = 1.28, p = 1.08 × 10^−5^) with full statistical power (1.00), indicating near-universal agreement regarding ease of use.

Commercial cap participants (n = 7) agreed that the cap showed general usability, as all averaged responses were above five (Fig. 9). The responses that were deemed to be significantly above the threshold include that participants felt the cap stayed in place and did not shift during the session (M = 8.86, SD = 0.900, p = 7.81 × 10^−3^). Participants also reported that they would feel comfortable wearing the cap for longer sessions and that the cap did not distract from the study (M = 8.71, SD = 1.80, p = 0.0156).

Willingness-based responses were similarly positive, with participants indicating they would feel comfortable recommending the cap to a friend (M = 8.86, SD = 0.690, p = 7.81 × 10^−3^) and would be willing to wear the cap again (M = 8.00, SD = 1.92, p = 0.0156).

Participants also reported that the cap was easy to put on and adjust to fit their heads (M = 9.29, SD = 0.756, p = 7.81 × 10^−3^).

In comparing the caps, the commercial cap showed higher average ratings than the custom cap in two questions related to usability. Commercial cap participants rated that the cap stayed in place more strongly than the custom cap, and this finding is reinforced by the average motion results between the two caps from motion resistance testing (Fig. 4). Participants also rated the commercial cap as easier to put on and adjust to fit their head, though by a slim margin (M = 9.22 for custom; M = 9.29 for commercial).

However, we found only one statistically significant relationship via Mann-Whitney testing, which is that participants were more willing to wear the custom cap again in comparison to the commercial cap (p = 0.0418). This significant relationship, and the fact that the custom cap produced a higher average for four of the six usability questions further, supports the custom cap being not only easy to deploy but also broadly acceptable for repeated and extended use. High willingness-to-wear-again and comparatively higher rating for willingness to wear longer (though not significant) underscores general user acceptance, which addresses the initial motivation for the custom cap design and is an essential factor for clinical translation.

## 4. Conclusion

In this study, we introduced and validated a novel, custom fNIRS cap designed specifically for prolonged use in the operating room. We demonstrated that the cap, over an extended duration, can maintain both mechanical stability and high signal quality without reliance on a traditional chinstrap for stabilization. Our results validate the cap both objectively (through motion resistance, signal quality values and captured hemodynamic trends) and subjectively around user comfort, discomfort and usability via human factors questionnaire.

The custom cap showed motion resistance in both x- and y-directions, as results remained significantly below predefined tolerances with particularly robust horizontal stability. It also showed strong signal quality throughout the procedure with the vast majority of channels showing higher SNR and over half of the channels showing high-quality SCI values. During breath holding, the expected increase in HbR and decrease in HbO were observed across participants. HbT, however, decreased during breath-holding, which deviates from the expected responses reported in previous literature [39,40]. This decrease, which we hypothesize resulted from the lack of an expiration event preceding the breath holding, was observed across both cap groups, suggesting that the unexpected HbT response was not specific to the custom cap design.

The custom cap was rated highly in terms of user-reported comfort, minimal perceived discomfort, and strong usability ratings. Further, the reported high willingness to wear again and for longer sessions supports the feasibility of using the custom cap for extended clinical or operational applications.

The custom cap was successfully implemented across a sample of participants with varied demographics (age, gender, race/ethnicity) and with five available cap sizes allowing cap selection based on individual fit. A limitation of the present study is that participant hair type and hairstyle were not systematically recorded, preventing direct evaluation of cap performance across these characteristics. While not evaluated in this study, the custom cap was designed to be compatible with our previously published hair-clearing optode attachments (“Mini Combs”) [41], providing an approach for hair clearing across varied hair characteristics in future applications of the cap. Future work evaluating the cap in combination with the Mini Combs and systematically characterizing participant hair would further establish custom cap performance across a broader range of hair types and styles.

Future work should also extend the present validation through a longer duration study and with larger, balanced sample sizes between cap conditions. Monitoring periods >2 hours would allow cap performance and participant comfort to be evaluated over durations more representative of extended-use applications. Further, larger and more equal sample sizes between the custom and commercial caps would strengthen comparisons and allow the present findings to be evaluated across a broader participant population.

One limitation of the current cap design is that the external tightening mechanism restricts access to specific 10–10 optode locations. These restrictions include the prefrontal (Fp) and all anterior frontal (AF) except AFz, lateral frontal locations F7-F10, lateral central locations FT9-FT10, T9-T10, and P9-P10, and Oz in the posterior. While these restrictions do not prevent measurements across the broader frontal, temporal, parietal, and occipital regions, they limit layouts requiring prefrontal or specific peripheral 10–10 locations. Future iterations of the cap will explore alternative tightening approaches, including mechanisms integrated directly into the cap fabric thus eliminating external or overlapping designs, to reduce interference with optode placement and further increase flexibility in probe configuration.

On the same note, the custom cap showed greater variability in vertical motion compared to horizontal motion, likely related to the absence of a chinstrap and again highlighting the need for an improved circumferential tightening mechanism. While this iteration of the cap was still shown to resist motion with statistical significance, future iterations could improve the vertical robustness and trend the motion in the y-direction more closely to the commercial cap.

Responses to the optional, open-ended section of the human factors questionnaire identified increased optode discomfort during the supine posture block as a recurring theme. This feedback was reported for both the custom and commercial caps and was associated with participants resting their heads against optodes positioned over parts of the occipital region. Future considerations for the custom cap could include adding cushioning to the outer layer of the design or incorporating a cushioned accessory designed to better distribute pressure when optodes contact external surfaces, potentially improving participant comfort during prolonged reclined or supine applications.

The custom cap could be further adapted and evaluated for use in areas of growing interest in fNIRS research. The current iteration of the cap was designed and evaluated using optode locations based on the international 10–10 system and was not evaluated with high-density probe configurations. As high-density fNIRS continues to expand [42,43], future work should investigate adaptations of the cap design that accommodate the closer source-detector spacing and increased number of optodes required for high-density measurements. Evaluation of these configurations will be important for determining the applicability of the cap beyond the probe layouts available in the present study.

Naturalistic deployment of fNIRS represents another important area for future evaluation [4,44]. The present study evaluated the custom cap in a mock OR, providing initial evidence of its performance in a setting outside of traditional labs. Future testing should extend to environments such as homes, schools, and offices where differences in participant activity, monitoring duration, and environmental conditions may further impact performance.

The findings from evaluation of the current cap design support its suitability for prolonged monitoring in OR environments where stability and comfort are critical. The achieved results and open-source nature of the design make this a promising cap for use outside of the OR and in general research as well. To enable ready-access to the cap for researchers utilizing cw-fNIRS, the files associated with cap creation are available on our lab OSF page. The design files associated with the cap are also annotated in the Supplement 1.

## Supplemental information

Supplement 1 Supplemetary Materials Document https://doi.org/10.6084/m9.figshare.33372241

## Data Availability

The information required to reproduce the cap evaluated in this study can be found at [45]. Data underlying the results presented in this paper are not publicly available at this time but may be obtained from the authors upon reasonable request due to ethical considerations and Institutional Review Board approval.

## Supplemental document

See Supplement 1 for supporting content.
