# Supplementary material for: Neckline-free open-source cap design and improved motion resistance for perioperative fNIRS procedures
Source: Biomed Opt Express. 2026 Sep 10;17(10):5077–96. doi: 10.1364/BOE.605391 (PMC13671698; doi:10.1364/BOE.605391)
Supplement: Supplementary file 1 [file boe-17-10-5077-s001.pdf]

Supplemental Document

Biomedical Optics EXPRESS

Neckline-free open-source cap design and improved motion resistance for perioperative fNIRS procedures: supplement

SETH BOLTON CRAWFORD,[1,2] SHIMRA JAIME FINE,[3] KYLA INEZ COVATO,[1,2] CAROLINE JOYCE CAVENESS,[1,4] AND AUDREY K. BOWDEN[1,2,5,*]

[1] *Vanderbilt Biophotonics Center, Vanderbilt University, Nashville, TN 37232, USA*
[2] *Department of Biomedical Engineering, Vanderbilt University, Nashville, TN 37232, USA*
[3] *Fine Soft Goods Consulting, Nashville, TN 37076, USA*
[4] *Department of Mechanical Engineering, Vanderbilt University, Nashville, TN 37232, USA*
[5] *Department of Electrical Engineering, Vanderbilt University, Nashville, TN 37232, USA*
[*] *a.bowden@vanderbilt.edu*

This supplement published with Optica Publishing Group on 10 September 2026 by The Authors under the terms of the Creative Commons Attribution 4.0 License in the format provided by the authors and unedited. Further distribution of this work must maintain attribution to the author(s) and the published article's title, journal citation, and DOI.

Supplement DOI: https://doi.org/10.6084/m9.figshare.33372241

Parent Article DOI: https://doi.org/10.1364/BOE.605391

TITLE OF PRIMARY MANUSCRIPT: A NECKLINE-FREE OPEN-SOURCED CAP DESIGN AND IMPROVED MOTION RESISTANCE FOR PERIOPERATIVE FNIRS PROCEDURES

1. Cap Fabrication

The files needed to fabricate the custom cap design can be found on our lab OSF page.

To access the files, navigate to the 'Files' path in OSF directory. We recommend opening the 'Materials and Links' folder first, as this provides you with the list of materials and links for purchasing needs to fabricate the caps (materialsList.xlsx). Once you have your materials, open the 'Fabrication Files' folder. Within that folder are five subfolders labeled with cap sizing information for extra small (XS), small (S), medium (M), large (L), and extra-large (XL) designs. Within each of these subfolders, there are two files: one for producing the inner or neoprene layer panels for the selected size and one for producing the outer or monofilament layer panels. All panels are designed in the same way but are of different sizes.

Within the 'Fabrication Files' folder is also a .pdf file named 'SewingInstructions' and it provides a sequential overview of how to sew together the panels to create the cap. These materials are sufficient to fully replicate the design of the custom cap evaluated in this study.

2. Posture-wise Signal Quality Comparison

Table S1. Posture-wise signal quality data.

	Standing	Upright Sitting	Reclined	Supine
Custom Cap (n=23)				
SNR (dB)	14.84	13.48	15.41	17.3
SCI	0.547	0.434	0.328	0.524
Commercial Cap (n=7)				
SNR (dB)	15.46	13.88	12.38	16.2
SCI	0.720	0.613	0.420	0.448

3. Human Factors - Full Questionnaire

3.1 Likert Questions:

1. The cap felt comfortable when first placed on my head.
2. The cap remained comfortable throughout the entire session.
3. The cap was still comfortable at the end of the session.
4. The pressure from the cap felt evenly distributed across my scalp.
5. The pressure from the embedded nodes/sensors was comfortable.
6. I felt hot or sweaty while wearing the cap.
7. The cap felt lightweight and did not cause head or neck fatigue.
8. The cap was easy to put on and adjust to fit my head properly.
9. The cap caused itchiness or irritation.
10. I would be willing to wear this cap again.
11. I would feel comfortable recommending this cap/study to a friend.

12. I felt restricted or tense while wearing the cap.
13. The cap contributed to feelings of mental fatigue.
14. The cap stayed in place and did not shift during the session.
15. The cap did not distract me or interfere with my ability to complete tasks.
16. The cap produced distracting sounds or sensations during use.
17. I would feel comfortable wearing this cap for a longer session (e.g., >1 hour).
18. Overall, I was satisfied with the comfort and fit of the cap.

3.2 Open Ended Questions:

1. What specific part(s) of the cap—if any—felt uncomfortable?
2. Did you experience any pressure points irritation from the cap or its sensors? If so, please describe.
3. Do you have any suggestions for improving the comfort or fit of the cap?
4. Was there anything unexpected about your experience wearing the cap—either positive or negative?
5. Is there anything else you would like to share about your experience wearing the cap?
